# The transcriptomic response of *Streptococcus pneumoniae* following exposure to cigarette smoke extract

**DOI:** 10.1038/s41598-018-34103-5

**Published:** 2018-10-24

**Authors:** Sam Manna, Alicia Waring, Angelica Papanicolaou, Nathan E. Hall, Steven Bozinovski, Eileen M. Dunne, Catherine Satzke

**Affiliations:** 1 Pneumococcal Research, Murdoch Children’s Research Institute, Infection and Immunity, Parkville, 3052 Australia; 2 Chronic Infectious and Inflammatory Disease Programme, School of Health & Biomedical Sciences, RMIT University, Bundoora, 3083 Australia; 3 Department of Animal, Plant and Soil Sciences, La Trobe University, Melbourne, Victoria, 3086 Australia; 4 Department of Paediatrics, The University of Melbourne, Parkville, 3052 Australia; 5 Department of Microbiology and Immunology, The University of Melbourne at the Peter Doherty Institute for Infection and Immunity, Parkville, 3010 Australia

## Abstract

Exposure to cigarette smoke is a risk factor for respiratory diseases. Although most research has focused on its effects on the host, cigarette smoke can also directly affect respiratory pathogens, in some cases enhancing virulence. *Streptococcus pneumoniae* (the pneumococcus) is a leading cause of community-acquired pneumonia worldwide, however data on the effects of cigarette smoke on the pneumococcus are sparse. Using RNA-seq, we show that pneumococci exposed to cigarette smoke extract in a concentrated acute exposure *in vitro* model initiate a ‘survival’ transcriptional response including the upregulation of detoxification enzymes, efflux pumps and osmoregulator transporters, as well as the downregulation of fatty acid and D-alanyl lipoteichoic acid biosynthesis genes. Except for the downregulation of the pneumolysin gene, there were no changes in the expression of major virulence factors following exposure to cigarette smoke. Compared to unexposed pneumococci, smoke-exposed pneumococci did not exhibit any changes in viability, adherence, hydrophobicity or cell lysis susceptibility. In this study, we demonstrate that pneumococci adapt to acute noxious cigarette smoke exposure by inducing a gene expression signature that allows the bacteria to resist its harmful effects.

## Introduction

Exposure to cigarette smoke increases susceptibility to respiratory infection due to the vast array of chemicals that irritate the airways and cause cell injury, lung inflammation, and reduced lung function^1,2^. These effects are also observed in young children that are commonly exposed to second-hand cigarette smoke, with infants living in households with smoking family members exhibiting a higher risk of lower respiratory tract infection^2,3^. Although damage to the host plays a critical role in the increased risk of respiratory infections associated with cigarette smoke exposure, emerging data suggest that other factors may contribute such as the effects of smoke on the pathogen itself. Bacteria can rapidly sense and respond to changes in the environment, including exposure to chemicals such as those in cigarette smoke^4,5^.

The direct effects of cigarette smoke have been investigated in multiple respiratory pathogens. Cigarette smoke exposure enhances biofilm formation by *Staphylococcus aureus*^6^, *Streptococcus gordonii*^7^, *Pseudomonas aeruginosa*^8^, *Candida albicans*^9^ and *Porphyromonas gingivalis‐S*. *gordonii* co-cultures^10^. Enhanced biofilm formation is often caused by smoke-induced changes in the expression of genes that facilitate biofilm formation (e.g. fimbrial and pilus genes, and transcriptional regulators of biofilm formation)^6,8,11^. In *S*. *aureus*, other phenotypes exhibited following cigarette smoke exposure include increased adherence to epithelial cells, reduced susceptibility to macrophage killing, and changes to the charge of the bacterial cell surface that reduce antimicrobial peptide affinity^6,12^.

*Streptococcus pneumoniae* (the pneumococcus) is a respiratory pathogen that is a common cause of community-acquired pneumonia. The risk of community-acquired pneumonia has been associated with cigarette smoking in adults, with positive relationships identified with number of cigarettes smoked per day and the use of unfiltered cigarettes^13^. Given these associations, it is not surprising that cigarette smoke is a well-known risk factor for pneumococcal disease^14^. For example, current smoking has been identified as an independent risk factor for septic shock complications during pneumococcal pneumonia^15^. In a colonization model, smoke-exposed mice that were intranasally infected with pneumococci exhibited reduced expression of TNF-α, CXCL-1 and CXCL-2 cytokines, suggesting cigarette smoke impairs the host response in the nasopharynx, facilitating the development of invasive disease^16^. Pneumococcal colonization of the upper respiratory tract is enhanced in mice exposed to cigarette smoke^17^. Pneumococcal burdens in the lungs of mice exposed to cigarette smoke were 4-fold greater at 24 hours post-infection compared to mice exposed to room air, which increased to 35-fold by 48 hours post-infection. Cigarette smoke-exposed mice also exhibited elevated production of cytokines IL-1β, 1L-6, IL-10 after pneumococcal challenge as well as impaired complement-mediated phagocytosis by alveolar macrophages^18^.

Few studies have investigated the direct impact of cigarette smoke on the pneumococcus. Pneumococcal cultures exposed to cigarette smoke condensate or black carbon exhibit enhanced biofilm formation^19,20^. Cigarette smoke also reduces the activity and expression of the major cytolytic toxin, pneumolysin^20,21^. However, the effect of cigarette smoke on pneumococcal gene expression, as well as on other phenotypes, remains unknown. Transcriptional data provide a broad understanding of how pathogens such as the pneumococcus can exploit hostile environments within a susceptible host. In this study, we aimed to investigate how pneumococcus responds to acute cigarette smoke exposure by assessing gene expression across the pneumococcal genome.

## Results and Discussion

We hypothesized that the chemicals in cigarette smoke would have profound effects on pneumococcal gene expression. To identify transcriptomic differences mediated by exposure to cigarette smoke, log-phase cultures of pneumococcal strain EF3030 (serotype 19F) were exposed to either THY media or THY treated with cigarette smoke (hereby referred to as CSE, cigarette smoke extract) using a short and concentrated acute exposure *in vitro* model. RNA was extracted from both cultures and RNA-Seq was performed to identify genes that were differentially expressed. Genes with a log_2_(fold change) of greater than 1 or less than −1 in CSE cultures relative to THY cultures and a false discovery of <0.05 were considered significantly differentially expressed in cigarette smoke.

**Figure 1:**
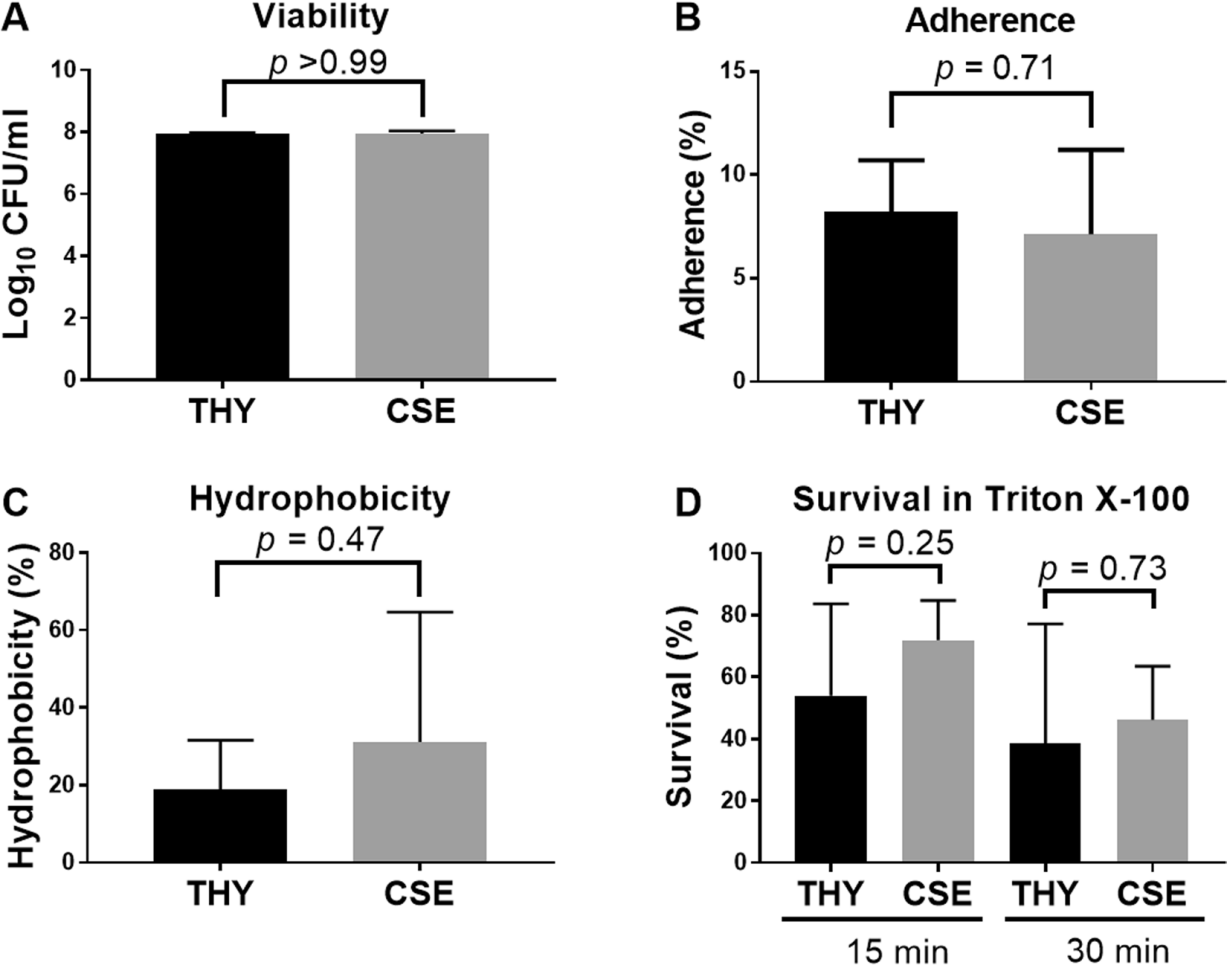
Effects of cigarette smoke exposure on pneumococcal phenotypes. Log-phase cultures of EF3030 were incubated in CSE or THY media for 45 min. Following incubation, cultures were assayed for (**A**) viability, (**B**) adherence to A549 lung epithelial cells, (**C**) hydrophobicity and (**D**) sensitivity to treatment with 0.005% Triton X-100. Data in panel A are from three independent experiments, presented as the median with the interquartile range, and analyzed by Mann-Whitney test. All other data (panels B–F) are from ≥3 independent experiments, presented as the mean with standard deviation and analyzed by unpaired t test.

Following incubation of pneumococcal cultures in CSE, 59 genes were upregulated compared to the THY control (Table 1). These include genes involved in competence, vitamin B6 synthesis and sugar catabolism. Other genes that were upregulated after CSE-exposure included glyoxalase (SPCG_RS00380), which removes methylglyoxals and other aldehydes (both of which can induce oxidative cell damage when accumulated to high levels^22^). Multiple oxidoreductase-encoding genes (SPCG_RS07925, SPCG_RS08280, SPCG_RS02990, SPCG_RS09075, SPCG_RS07475 and SPCG_RS05800) were also upregulated in CSE. Oxidoreductases also play a role in the removal of glyoxals^22^. In addition, multiple metal ion transporters and metal-dependent transcriptional regulators including a zinc efflux (SPCG_RS09490, SPCG_RS09485 and SPCG_RS09480) and copper efflux operon (SPCG_RS03545, SPCG_RS03550, SPCG_RS03555) were upregulated in CSE. Cigarette smoke contains an array of toxic substances including methylglyoxals and glyoxals, as well as metals such as zinc and copper^4,5^. Interestingly, zinc transporter *czcD* (SCPG_RS09490) is also upregulated in the presence of cadmium^23^, which is also present in cigarette smoke^5^. The intracellular accumulation of cadmium is also associated with oxidative stress by interfering with manganese uptake^23^. The upregulation of these genes in pneumococcus is likely to detoxify or remove these potentially harmful substances from the cell and combat oxidative stress. Interestingly, viable counts of pneumococci incubated in either THY or CSE did not exhibit any difference in viability (Fig. 1A, Supplementary Fig. S1), suggesting the upregulation of these genes may help the bacteria to survive in the presence of these substances.

**Table 1:** Pneumococcal genes upregulated following 45 min incubation in cigarette smoke-treated THY (CSE) media relative to cultures incubated in THY media.

Functional group or gene	Description	log_2_ (fold change)	FDR
**Competence/competence induced cell lysis**			
***SPCG_RS10625***	Competence protein ComGC	1.81	0.048
SPCG_RS04840	Late competence protein ComEA, DNA receptor	1.43	0.012
SPCG_RS10610	Competence protein ComGF	1.37	0.044
SPCG_RS11465	Choline-binding protein D CbpD	1.29	0.033
SPCG_RS06370	DNA protecting protein DprA	1.27	0.041
SPCG_RS04845	Late competence protein ComEC, DNA transport	1.17	0.049
**Two-component regulatory systems**			
***SPCG_RS10300***	Two-component system transcriptional response regulator 11	2.21	0.004
***SPCG_RS10305***	Two-component system sensor histidine kinase 11	2.12	0.006
**Nucleotide transport/biosynthesis**			
SPCG_RS01145	Anaerobic ribonucleoside-triphosphate reductase activating protein NrdG	1.92	0.022
SPCG_RS09500	Nicotinamide mononucleotide transporter PnuC	1.89	0.006
SPCG_RS01150	Phosphoribulokinase	1.73	0.038
SPCG_RS09080	NADPH-dependent 7-cyano-7-deazaguanine reductase QueF	1.49	0.011
**Glycine betaine transport**			
SPCG_RS09515	Hypothetical protein	2.75	0.008
***SPCG_RS09520***	MarR family transcriptional regulator	2.70	0.008
SPCG_RS09510	Glycine/betaine ABC transporter ATP-binding protein ProV	2.57	0.011
SPCG_RS09505	Glycine/betaine ABC transporter permease ProWX	2.41	0.007
**Sugar catabolism**			
SPCG_RS09465	Galactose-1-phosphate uridylyltransferase GalT2	2.22	0.003
SPCG_RS09700	Sugar ABC transporter substrate-binding protein MsmE	1.89	0.038
SPCG_RS03585	6-phospho-beta-glucosidase	1.82	0.038
SPCG_RS09705	Alpha-galactosidase Aga	1.72	0.042
SPCG_RS01445	Glutamine-fructose-6-phosphate aminotransferase GlmS	1.54	0.005
SPCG_RS09680	Sucrose phosphorylase GtfA	1.33	0.037
SPCG_RS09470	Galactokinase GalK	1.26	0.007
**Vitamin B6 biosynthesis**			
SPCG_RS07540	Pyridoxal 5′-phosphate synthase subunit PdxS	1.67	0.004
SPCG_RS07535	Pyridoxal 5′-phosphate synthase subunit PdxT	1.41	0.007
**Acetolactate synthesis**			
SPCG_RS02285	Acetolactate synthase, large subunit IlvB	1.34	0.006
SPCG_RS02290	Acetolactate synthase, small subunit IlvH	1.00	0.044
**Oxidoreductases**			
SPCG_RS07925	NAD(P)H-dependent FMN reductase	3.32	0.000
SPCG_RS08280	NADH-flavin reductase	2.46	0.034
SPCG_RS02990	NAD(P)H-flavin reductase	2.35	0.004
SPCG_RS09075	Thioredoxin	2.02	0.004
SPCG_RS07475	Thioredoxin-disulfide reductase	1.97	0.004
SPCG_RS05800	Glutaredoxin	1.14	0.048
**Metal transport**			
SPCG_RS09480	Zn-dependent alcohol dehydrogenase AdhB	4.30	0.0004
SPCG_RS09485	MerR family transcriptional regulator	3.97	0.003
***SPCG_RS09490***	Cation transporter, CzcD	3.50	0.004
SPCG_RS03555	Copper-translocating P-type ATPase, CopA	2.46	0.004
SPCG_RS03550	Cupredoxin domain-containing protein, CupA	2.42	0.004
SPCG_RS03545	CopY/TcrY family copper transport transcriptional repressor, CopY	2.41	0.004
***SPCG_RS00385***	Metal-sensitive transcriptional repressor, FrmR family	2.36	0.013
SPCG_RS01895	Peptide ABC transporter ATP-binding protein	1.31	0.006
**Adherence**			
SPCG_RS06505	Choline-binding protein A, PcpA	1.34	0.046
**Other genes**			
***SPCG_RS00380***	Glyoxalase	4.46	0.003
SPCG_RS04530	Chlorohydrolase, predicted pseudogene	3.33	0.005
SPCG_RS11630	Membrane protein YhgE, phage infection protein (PIP) family	2.65	0.006
SPCG_RS09070	DUF4649 domain-containing protein	2.39	0.009
SPCG_RS10310	ABC transporter permease	2.05	0.021
SPCG_RS10320	Hypothetical protein	1.94	0.022
SPCG_RS10315	ABC transporter ATP-binding protein	1.92	0.007
SPCG_RS01140	GNAT family acetyltransferase	1.75	0.044
***SPCG_RS09065***	MarR family transcriptional regulator	1.50	0.004
SPCG_RS10895	Predicted transcriptional regulator, pseudogene	1.46	0.048
SPCG_RS07525	ABC-type lipoprotein export system, ATPase component	1.33	0.049
SPCG_RS00665	tRNA-specific 2-thiouridylase MnmA	1.18	0.015
SPCG_RS03595	Sodium-dependent transporter	1.13	0.008
SPCG_RS10255	Putative cell surface protein with DUF1542 domain	1.10	0.019
SPCG_RS10750	tRNA-Ile	1.08	0.008
SPCG_RS10445	Permease	1.07	0.037
SPCG_RS10695	MATE family efflux transporter	1.02	0.037

SPCG number is the locus tag ID for each gene in the CGSP14 genome sequence, which was used to map the EF3030 RNA reads from two independent cultures. Only differentially expressed genes are shown, defined as a log_2_(fold change) of greater than 1 or less than −1 and a false discovery rate (FDR) of <0.05. Genes in bold and italics were validated by qRT-PCR.

The glycine betaine ABC transporter operon encodes proteins that respond to osmotic stress. The gene products in this operon play a role in the accumulation of compatible solutes such as proline and glycine betaine, which are essential for osmoregulation^24–26^. The genes in this putative operon, including a MarR family transcriptional regulator (SPCG_RS09520), hypothetical protein (SPCG_RS09515), glycine betaine ABC transporter ATP-binding protein, ProV (SPCG_RS09510) and a glycine betaine ABC transporter permease, ProWX (SPCG_RS09505), were all upregulated after CSE exposure (Table 1). These genes were also upregulated during the acid tolerance response of pneumococci to low pH conditions^27^. In our experiments, cigarette smoke treatment of THY media lowered the pH by ~0.8 (mean pH of CSE media 7.09 ± 0.13 compared to 7.91 ± 0.20 for THY), which is lower than the optimal pH for pneumococci^27,28^. Martín-Galiano *et al*. proposed a relationship between the pneumococcal acid tolerance response, and other stress responses including oxidative and osmotic stress^27^. As such, the changes in pneumococcal gene expression in response to cigarette smoke may be mediated via direct exposure to chemicals in cigarette smoke and/or changes in physiological pH.

Smoke exposure enhances biofilm formation in bacterial pathogens including *P*. *aeruginosa*, *S*. *gordonii* and *S*. *aureus*^6–8^. Pneumococcal cultures exposed to cigarette smoke condensate also exhibit augmented biofilm formation^20^. In our study, *pcpA*, which encodes a choline-binding protein, was upregulated in CSE. PcpA contributes to biofilm formation, supported by the diminished biofilm formation phenotype observed in *pcpA* mutants^29^. The upregulation of *pcpA* in CSE we identified in this study is consistent with published data on increased biofilm formation exhibited by pneumococcus following smoke exposure^20^ as well as previous reports on the upregulation of this gene during metal stress^30,31^. Some pneumococci contain a 7 kb insertion encoding a second copy of the *pspA* and *pcpA* genes^32^. This is also the case in EF3030, the strain used in this study. Interestingly, only one *pcpA* gene (SPCG_RS06505) was upregulated in CSE. PcpA has also been reported to play a role in adherence to nasopharyngeal and lung epithelial cells^33^. Although, CSE-treated pneumococcal cultures exhibit an upregulation of *pcpA*, no increase in adherence to A549 lung epithelial cells was observed (Fig. 1B). This may be because *pcpA* was the only adhesin that was upregulated in CSE. In the absence of changes in expression of other major adhesins (e.g. PspA), this may not be sufficient to increase adherence. In addition, cigarette smoke exposure of A549 cells increases the expression of PAFR (platelet activating factor receptor), which enhances pneumococcal adhesion^34^. In our study, A549 cells were not exposed to cigarette smoke. Taken together, these findings on the augmented pneumococcal adherence phenotype described previously are driven more by the effects of cigarette smoke on the host rather than on the pathogen itself.

An important component of biofilms is extracellular DNA. In pneumococci, this is partially mediated by bacterial fratricide, whereby non-competent pneumococci are killed by their competent neighbors^35,36^. The DNA released from lysed pneumococci can not only be taken up by competent pneumococci, but serves as an important component of the biofilm matrix. Competence genes upregulated in CSE include *cbpD*, which encodes choline-binding protein D (SPCG_RS11465), a murein hydrolyase that attacks non-competent pneumococci resulting in the release of DNA into the environment^37,38^. Despite this, *cbpD* mutants do not exhibit a diminished biofilm formation phenotype^29^, although a reduced colonization phenotype is observed^39^. Like *cbpD*, the other competence genes upregulated in CSE were from the late stage. These were involved in various stages of the DNA uptake process including type-IV-like pilus formation (*comGC*, SPCG_RS10625 and *comGF*, SPCG_RS10610), binding of extracellular DNA (*comEA*, SPCG_RS04840) and its transport into the cell (*comEC*, SPCG_RS04845), as well as its loading on the RecA recombinase (*dprA*, SPCG_RS06370) so it can undergo homologous recombination with the chromosome. Pneumococcal fratricide is important for genetic exchange within the context of a biofilm^40^, and exogenous addition of competence stimulating peptide (the inducer of competence) enhanced biofilm formation^41^. Pneumococci cultured within a biofilm exhibit upregulation of competence genes compared to planktonic cells^42^. These competence genes are constitutively upregulated in pneumococcal biofilms cultured under *in vitro* conditions simulating the environment of the nasopharynx (e.g. lower temperature of 32–34 °C and restricted nutrient accessibility)^42^. The upregulation of competence genes in response to CSE may explain the enhanced biofilm formation observed previously by Cockeran *et al*.^20^. In addition, the upregulation of competence genes can occur during stress conditions such as antibiotic stress and DNA damage^43–45^. Together with the array of metals and chemicals found in cigarette smoke, it is likely that the upregulation of competence genes in response to CSE is stress-related. However, future studies should investigate whether cigarette smoke could enhance DNA uptake and recombination of pneumococci from other pneumococci as well other bacteria within the nasopharynx.

In the differential gene expression analysis, 63 genes were significantly downregulated in CSE (Table 2). Among these genes, a substantial proportion is predicted to result in changes to the pneumococcal cell surface, including 15 genes involved in fatty acid biosynthesis (Table 2). Fatty acids are an important component in phospholipids that make up the lipid bilayer of cell membranes. Bacteria are capable of controlling membrane lipid homeostasis by regulating the expression of genes involved in fatty acid biosynthesis, which affects various membrane functions such as permeability^46^. In *Streptococcus mutans*, deletion of the fatty acid isomerase gene *fabM* results in significant changes to membrane composition and enhanced ATPase activity, proton permeability and phosphotransferase activity^47^. The pneumococcal homolog of *fabM* (SPCG_RS02150) was among the fatty acid biosynthesis genes that were downregulated. Some ATPases and transporters were also upregulated following CSE exposure (e.g. SPCG_RS10310, SPCG_RS10315 and SPCG_RS07525, Table 1). The downregulation of fatty acid biosynthesis genes in CSE-exposed cells suggested that cigarette smoke may induce changes in the hydrophobicity of the pneumococcal cell surface. However, when pneumococcal hydrophobicity was evaluated, no difference between CSE and THY cultures was observed (Fig. 1C). Cigarette smoke has been shown to increase hydrophobicity in *S*. *aureus*^12^. It is therefore plausible that cigarette smoke would be acting in a similar manner on pneumococcus. However, unlike *S*. *aureus*, pneumococci downregulate fatty acid biosynthesis genes, possibly as a mechanism to maintain cell surface hydrophobicity, rather than allowing it to increase. Previous work on the effects of cigarette smoke on *S*. *aureus* used a non-encapsulated strain^12^. This is in contrast to our study in which EF3030 produces a serotype 19F capsular polysaccharide. It is plausible that the pneumococcal extracellular capsular polysaccharide may provide a physical barrier that masks the effects of cigarette smoke on cell surface hydrophobicity. Future studies could explore this area further by testing the hydrophobicity of isogenic capsule mutants.

**Table 2:** Pneumococcal genes downregulated following 45 min incubation in cigarette smoke-treated THY (CSE) media relative to cultures incubated in THY media.

Functional group or gene	Description	log_2_ (fold change)	FDR
**Fatty acid biosynthesis**			
***SPCG_RS02210***	Acetyl-CoA carboxylase carboxyl transferase subunit alpha AccA	−3.64	0.003
SPCG_RS02205	Acetyl-CoA carboxylase carboxyl transferase subunit beta AccD	−3.59	0.003
SPCG_RS02200	Acetyl-CoA carboxylase biotin carboxylase subunit AccC	−3.34	0.004
SPCG_RS02190	Acetyl-CoA carboxylase biotin carboxyl carrier protein subunit AccB	−3.32	0.004
SPCG_RS02195	3-hydroxyacyl-ACP dehydratase FabZ	−3.18	0.005
SPCG_RS02185	3-oxoacyl-ACP synthase FabF	−3.11	0.004
SPCG_RS02180	3-ketoacyl-ACP reductase FabG	−2.92	0.006
SPCG_RS02150	Enoyl-CoA hydratase FabM	−2.82	0.026
SPCG_RS02175	ACP S-malonyltransferase FabD	−2.76	0.011
SPCG_RS03620	Fatty acid-binding DegV family protein	−2.59	0.006
SPCG_RS02170	Enoyl-[acyl-carrier-protein] reductase FabK	−2.10	0.036
SPCG_RS02215	Acetyl-CoA carboxylase	−2.06	0.007
SPCG_RS02160	3-oxoacyl-ACP synthase III FabH	−1.62	0.011
SPCG_RS02165	Acyl carrier protein, AcpP	−1.56	0.005
***SPCG_RS02155***	MarR family transcriptional regulator FabT	−1.45	0.011
**Pyrimidine biosynthesis**			
SPCG_RS03420	Orotate phosphoribosyltransferase PyrE	−2.54	0.003
SPCG_RS03415	Orotidine 5′-phosphate decarboxylase PyrF	−2.30	0.006
SPCG_RS06430	Bifunctional pyrimidine operon transcriptional regulator/uracil phosphoribosyltransferase PyrR	−1.83	0.004
SPCG_RS06420	Carbamoyl-phosphate synthase small chain CarA	−1.82	0.004
SPCG_RS06425	Aspartate carbamoyltransferase PyrB	−1.80	0.004
SPCG_RS04890	Dihydroorotate dehydrogenase PyrD	−1.73	0.004
SPCG_RS06415	Carbamoyl-phosphate synthase large chain CarB	−1.52	0.005
SPCG_RS06470	Uracil permease PyrP	−1.44	0.010
SPCG_RS04885	Dihydroorotate dehydrogenase PyrDII	−1.34	0.012
**Virulence**			
***SPCG_RS09950***	Pneumolysin Ply	−3.39	0.004
**D-alanyl-lipoteichoic acid biosynthesis**			
***SPCG_RS11330***	D-alanyl-lipoteichoic acid biosynthesis protein DltB	−1.51	0.031
SPCG_RS11320	D-alanyl-lipoteichoic acid biosynthesis protein DltD	−1.47	0.025
SPCG_RS11325	D-alanine-poly(phosphoribitol) ligase subunit 2 DltC	−1.46	0.007
SPCG_RS11340	D-alanyl-lipoteichoic acid biosynthesis protein DltX	−1.40	0.011
SPCG_RS11335	D-alanine-poly(phosphoribitol) ligase subunit 1 DltA	−1.24	0.046
**Carbohydrate transport and metabolism**			
SPCG_RS07255	Glucosamine-6-phosphate deaminase NagB	−1.99	0.049
SPCG_RS10975	Glycogen/starch/alpha-glucan family phosphorylase MalP	−1.77	0.046
SPCG_RS00515	Glycosyltransferase	−1.64	0.042
SPCG_RS00520	Polysaccharide biosynthesis protein	−1.48	0.037
SPCG_RS04895	Endo-beta-N-acetylglucosaminidase LytB	−1.40	0.004
SPCG_RS08595	Carbohydrate ABC transporter permease	−1.13	0.044
SPCG_RS09625	Phosphotransferase system trehalose transporter subunit IIABC TreP	−1.07	0.014
**Cell wall/membrane biogenesis**			
SPCG_RS00545	Peptidoglycan-binding protein LysM	−1.76	0.004
SPCG_RS04610	ABC-type lipoprotein export system, ATPase component	−1.40	0.019
SPCG_RS10170	Membrane protein insertase YidC	−1.16	0.021
**Lipid transport and metabolism**			
SPCG_RS04125	Glycerol-3-phosphate acyltransferase	−1.27	0.006
SPCG_RS08265	1-acyl-sn-glycerol-3-phosphate acyltransferase	−1.16	0.046
**Proteases**			
SPCG_RS02225	CAAX protease self-immunity protein	−2.28	0.004
SPCG_RS01770	ATP-dependent Clp protease ATP-binding protein ClpL	−2.08	0.037
SPCG_RS11675	Serine protease HtrA	−1.23	0.044
SPCG_RS11690	Membrane proteinase	−1.16	0.046
**Other genes**			
SPCG_RS09955	Hypothetical protein	−2.94	0.004
SPCG_RS02220	Membrane protein	−2.23	0.005
SPCG_RS09960	Hypothetical protein	−2.80	0.006
SPCG_RS09965	DUF4231 domain-containing protein	−2.20	0.048
SPCG_RS10500	Bifunctional acetaldehyde-CoA/alcohol dehydrogenase	−1.91	0.024
SPCG_RS08740	Sodium ABC transporter permease	−1.76	0.029
SPCG_RS04615	FtsX-like ABC transporter permease	−1.67	0.007
SPCG_RS03430	Hypothetical protein	−1.57	0.015
SPCG_RS11680	Chromosome partitioning protein ParB	−1.54	0.038
SPCG_RS08185	Hypothetical protein	−1.42	0.006
SPCG_RS03900	DUF3270 domain-containing protein	−1.28	0.048
SPCG_RS03795	Hypothetical protein	−1.24	0.008
***SPCG_RS09945***	YebC/PmpR family DNA-binding transcriptional regulator	−1.24	0.044
SPCG_RS04600	Phage shock protein PspC (stress-responsive transcriptional regulator)	−1.20	0.038
SPCG_RS07515	Putative channel-forming cytolysin, hemolysin III family	−1.12	0.019
SPCG_RS08585	Oxidoreductase	−1.09	0.029
SPCG_RS05215	DUF1002 domain-containing protein	−1.00	0.038

SPCG number is the locus tag ID for each gene in the CGSP14 genome sequence, which was used to map the EF3030 RNA reads from two independent cultures. Only differentially expressed genes are shown, defined as a log_2_(fold change) of greater than 1 or less than −1 and a false discovery rate (FDR) of <0.05. Genes in bold and italics were validated by qRT-PCR.

Another cigarette smoke-driven effect on the pneumococcal cell surface with the potential to impact membrane permeability and molecule affinity is the downregulation of the *dlt* operon (SPCG_RS11320, SPCG_RS11325, SPCG_RS11330, SPCG_RS11335 and SPCG_RS11340). This operon encodes proteins involved in D-alanyl-lipoteichoic acid biosynthesis, which involves the addition of D-alanine onto teichoic acids in the cell wall. This reduces the negative charge of the cell surface, diminishing its affinity to cationic antimicrobial peptides (CAMPs) and promoting the evasion of CAMP-mediated killing^48^. Pneumococcal *dlt* mutants display increased susceptibility to CAMPs^48^, and upregulation of the *dlt* operon has been observed in response to the presence of CAMPs such as nisin and LL-37^49^. Interestingly, the lack of D-alanyl lipoteichoic acids (due to repression of the *dlt* operon) in CSE-exposed pneumococci would increase its negative charge. The changes at the pneumococcal cell surface raise an interesting question around how smoke-exposed pneumococci would behave in the context of the nasopharyngeal environment. The pneumococcal capsule is negatively charged, which facilitates pneumococcal colonization. This is because the capsule mediates electrostatic repulsion of the mucus lining of the upper respiratory tract, which is also negatively charged due high levels of sialic acid. This allows the bacterium to escape mucus-dependent clearance^50^. In our study, the putative CSE-induced increase in negative charge of the cell surface (from the downregulation of the *dlt* operon) would likely enhance colonization of the nasopharynx by a similar mechanism. Pneumococcal colonization of the upper respiratory tract is enhanced in mice exposed to cigarette smoke^17^. With multiple smoke-induced changes in pneumococcal gene expression that could impact colonization, it is plausible that the effects of smoke on the pneumococcus may contribute to this phenotype.

Two other genes involved in cell wall metabolism were also downregulated in CSE. These included *lytB* (endo-beta-N-acetylglucosaminidase, SPCG_RS04895) and *lysM* (peptidoglycan-binding protein, SPCG_RS00545). The differential expression of these genes could induce changes in the cell wall architecture, which may influence susceptibility to cell lysis and potentially influence the ability of the bacterium to persist within a host. We therefore assessed whether CSE exposure would impact susceptibility to cell lysis. However, no difference was observed in the ability of CSE and THY cultures to resist detergent-mediated lysis (Fig. 1D), suggesting the integrity of the cell wall has not been modified by CSE exposure.

Following exposure to CSE, the major virulence gene, *ply* (SPCG_RS09950), which encodes the cytolytic toxin pneumolysin, was downregulated (Table 2), a finding that is consistent with what has been described previously^20^. Although some genes with virulence associated roles in metal transport and adherence were differentially expressed in CSE, most major pneumococcal virulence factors (e.g. *lytA*, *pspA*, *nanA*, capsular polysaccharide biosynthesis genes)^51^ were not. Taken together with the previous work of Cockeran *et al*.^20^, our data suggest that exposure of pneumococcus to cigarette smoke promotes survival and biofilm formation.

**Figure 2:**
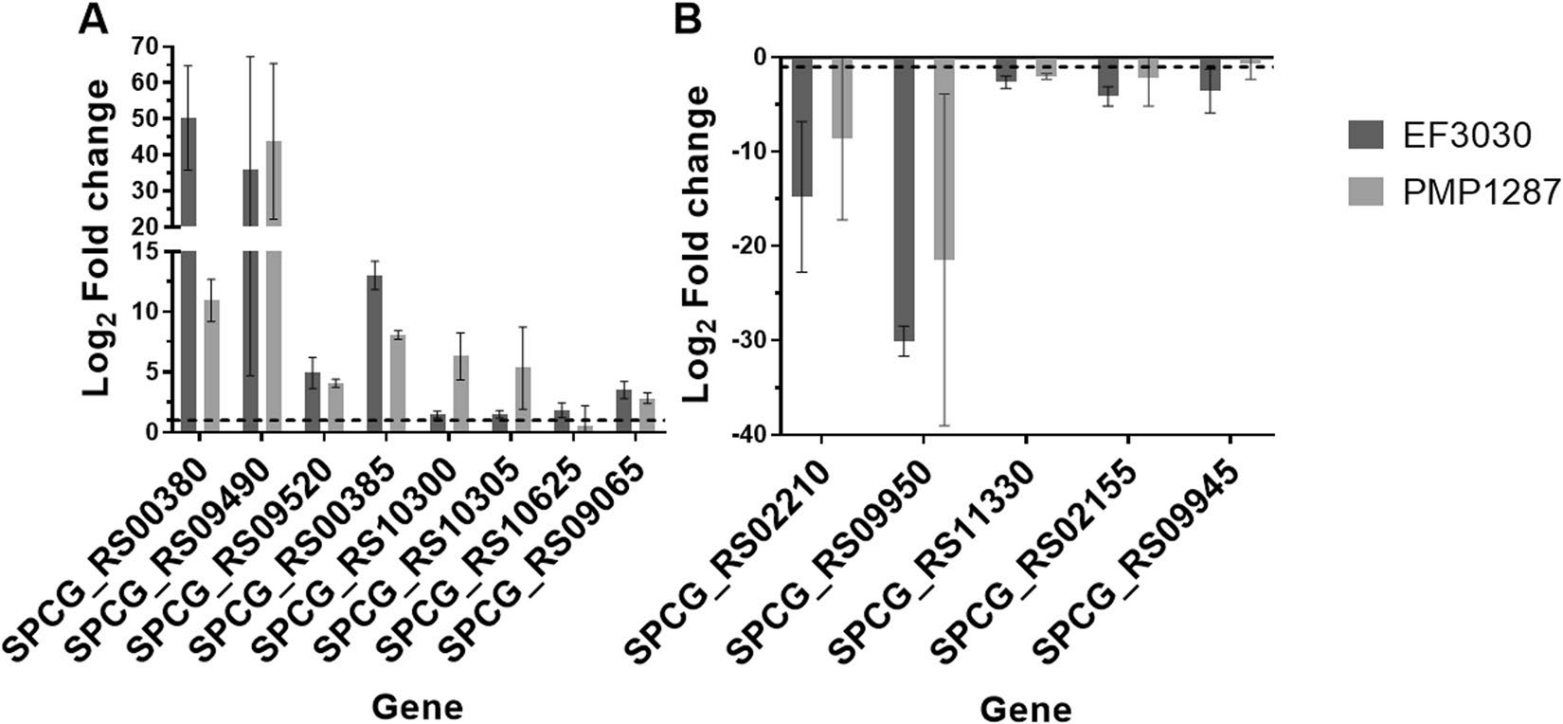
qRT-PCR analysis in strains EF3030 and PMP1287. Genes tested were a selection of 13 genes that were upregulated (**A**) and downregulated (**B**) using RNA-seq following exposure to CSE. Selected genes were; SPCG_RS00380 (glyoxalase), SPCG_RS09490 (*czcD*), SPCG_RS09520 (MarR family transcriptional regulator), SPCG_RS00385 (Metal sensitive transcriptional repressor), SPCG_RS10300 (two-component system transcriptional response regulator 11), SPCG_RS10305 (two-component system sensor histidine kinase 11), SPCG_RS10625 (*comGC*), SPCG_RS09065 (MarR family transcriptional regulator), SPCG_RS02210 (*accA*), SPCG_RS09950 (*ply*), SPCG_RS11330 (*dltB*), SPCG_RS02155 (*fabT*) and SPCG_RS09945 (YebC/PmpR family transcriptional regulator). Pneumococci were exposed to CSE or control media for 45 min prior to RNA extraction. Data were normalized to the *gyrA* gene using the 2−ΔΔCt method. Data are presented as the mean fold change ± standard deviation in cultures incubated in CSE relative to those incubated in THY control media (n = 3 independent experiments). Dotted line represents the biological significance threshold (log_2_ = 1 or −1). Confidence intervals are provided in Table S2.

To confirm whether the genes identified in RNA-Seq are indeed differentially expressed following CSE exposure, a subset of 13 genes (8 upregulated and 5 downregulated) that were differentially expressed in EF3030 CSE cultures by RNA-Seq were validated by quantitative reverse transcription polymerase chain reaction (qRT-PCR). The 13 genes were chosen to represent diverse functional categories including metal transport, competence, fatty acid biosynthesis, virulence, D-alanyl-lipoteichoic acid biosynthesis and transcriptional regulation. Consistent with the RNA-Seq findings, all 13 genes that were differentially expressed in CSE using RNA-Seq were also differentially expressed and in the same direction when assessed by qRT-PCR in EF3030 (Fig. 2).

We assessed the impact of CSE on gene expression in another pneumococcal strain (PMP1287, serotype 16F). Of the panel of selected genes, 11 were also differentially expressed in PMP1287 following exposure to cigarette smoke and in the same direction as EF3030 (Fig. 2), whilst two genes in the panel were not differentially expressed in PMP1287 after smoke exposure (SPCG_RS10625 and SPCG_RS09945). In addition to the downregulation of *ply*, Cockeran *et al*. also demonstrated that the histidine kinase and response regulator that comprise two-component regulatory system 11 (TCS11) were upregulated following exposure of a serotype 23F pneumococcal strain to cigarette smoke^20^. Our findings support this, as these three genes were also differentially expressed in both strains used in our study (Fig. 2, SPCG_RS10300, SPCG_RS10305 and SPCG_RS09950).

The role of TCS11 is not clear and the genes regulated by this system have not been described to date. Mutation of the histidine kinase and response regulator genes of TCS11 had no effect in a murine respiratory tract infection model, suggesting it does not play a role in virulence^52^. It has been postulated that TCS11 may be involved in responding to stress caused by environmental stimuli^53^, indicated by its upregulation following exposure to the antibiotic vancomycin^54^. The upregulation of TCS11 following CSE exposure supports this notion.

An interesting question is whether these changes in gene expression also occur within *in vivo* systems. The *in vitro* system is disadvantaged by the fact that it is not a physiologically relevant system and does not accurately reconstruct a pneumococcal carriage episode. Examining pneumococcal gene expression in colonized mice exposed to either cigarette smoke or air will determine whether the transcriptomic response observed in our study is also an *in vivo* occurrence. It will also allow the investigation of whether these changes are tissue-specific (e.g. comparing pneumococcal expression in the nasopharynx vs lung of smoke-exposed mice). To better understand how these changes relate to pneumococcal carriage and smoking behavior in humans, this mouse model can also be used to uncover if smoke-induced changes in pneumococcal gene expression are dependent on dose or exposure duration, which were only briefly investigated in the *in vitro* system of this study (Supplementary Figs S1 and S2). The results from our study suggest that the increased susceptibility to pneumococcal disease in smokers would unlikely be due to smoke-induced changes in the pathogen itself. However, it is still not clear whether this increased susceptibility is due to a synergistic combination of smoke-induced effects on both the pathogen and the host. For example, it is possible that pneumococci modify their gene expression profile for enhanced virulence by detecting and responding to the smoke-induced damage to the host (e.g. detection of key inflammatory agents produced by the host in response to cigarette smoke), which would not be detected using *in vitro* systems. A smoke-exposure animal model would be suited to answering this question. In addition, the results from our study demonstrate that pneumococci have the capacity to resist the harmful effects of cigarette smoke. This would allow pneumococci to survive in a smoke-exposed host environment, which together with a more susceptible host may play a significant role in the increased infection risk observed in smokers.

The work described in this study begins to elucidate the genetic basis underlying the behavior of pneumococcus in a cigarette smoke-exposed environment. To our knowledge, this is the first report evaluating the direct effects of cigarette smoke on a respiratory pathogen from a transcriptomic perspective and therefore provides novel insights into the global response of pneumococci to cigarette smoke exposure. These responses include; (1) an upregulation of genes facilitating the detoxification and removal of toxic compounds; (2) activation of genes responsive to physiological stress; (3) upregulation of genes involved in gene exchange and biofilm formation and; (4) repression of genes required for fatty acid biosynthesis and D-alanylation of lipoteichoic acids. These changes ensure survivability and adaptability of pneumococci in a cigarette smoke exposed environment. The phenotypes that were assessed (viability, adherence, hydrophobicity and lysis susceptibility) did not change upon CSE exposure, which may be due to rapid reversion of gene expression profile upon removal of the CSE stimulus at the time the phenotypes were investigated. Alternatively, it is possible that the phenotypes do not change as the transcriptomic changes in gene expression allow pneumococci to resist the harmful effects of CSE. Our data demonstrate that smoke exposure results in a gene expression signature of survivability and adaptability rather than directly impacting pneumococcal virulence. Future studies should focus on testing different lengths of exposure duration as well as concentration of CSE to determine if the same changes in gene expression are observed.

## Materials and Methods

### Preparation of cigarette smoke extract media

Cigarette smoke extract (CSE) medium was prepared using a method adapted from a previous study^55^. In brief, four 3R4F research grade cigarettes (Kentucky Tobacco Research & Development Centre, University of Kentucky) were bubbled through 10 ml of THY medium (3% [w/v] Todd-Hewitt broth, 0.5% [w/v] yeast extract). This produced the highest concentration of CSE without affecting pneumococcal viability (Supplementary Fig. S1) and was thereby considered to provide the strongest transcriptional response. This was performed using a smoke apparatus and syringe to draw the smoke into the medium. Cigarette smoke was drawn in 30 ml tidal volumes with a 20 sec break between draws. The control THY medium was prepared in the same manner except that air was drawn through the apparatus instead of cigarette smoke. Air controls were conducted in a chemical fume hood prior to preparation of CSE to ensure there is no contamination of control media with residual smoke. Media were filter sterilized before use with a 0.22 μm syringe-driven filter.

### Exposure of pneumococcal cultures to CSE

Pneumococcal strain EF3030^56^ (serotype 19F, multi-locus sequence type 43) or PMP1287 (serotype 16F, mutli-locus sequence type 3117) was inoculated in THY media and incubated at 37 °C, 5% CO_2_ to an OD_600_ of 0.1–0.2. Bacteria were then harvested in 3 ml aliquots by centrifugation (1820 × *g* for 2 min) and the supernatant removed. Pellets were resuspended in 3 ml of THY or CSE and incubated for 45 min. This time point was chosen based on experiments examining expression of the *ply* operon following incubation of pneumococcal cultures in CSE (Supplementary Fig. S2). This operon was chosen as it included the *ply* gene, which has been demonstrated to be differentially expressed in response to cigarette smoke previously^20^. For the enumeration of pneumococci following CSE exposure, cultures were serially diluted in 0.85% saline, plated on horse blood agar plates and incubated overnight at 37 °C, 5% CO_2_. Additionally, 0.5 ml aliquots of pneumococcal cultures were mixed with 1 ml RNAprotect Bacteria Reagent (Qiagen), incubated at room temperature for 5 min and stored at −80 °C for RNA extraction.

### RNA extraction

Extraction of RNA from pneumococci was performed using the RNeasy Mini Kit (Qiagen) according to the manufacturer’s instructions with the incorporation of an additional lysis step. In summary, bacterial preparations that were stored in RNAprotect Bacteria Reagent (Qiagen) (as described above) were thawed and incubated at 37 °C for 15 min in 200 µl lysis buffer containing 600 µg/ml lysozyme (Sigma-Aldrich), 15 µg/ml mutanolysin (Sigma-Aldrich), 1U/µg SUPERase In RNase Inhibitor (Ambion) and 400 µg/ml Proteinase K (Qiagen) in TE buffer. The rest of the procedure was conducted as per the RNeasy Mini Kit instructions. An on-column DNase treatment was performed by the addition of 2727 Kunitz U/ml DNase I in buffer RDD, (Qiagen), and incubated at room temperature for 15 min prior to washing and elution in 20 µl RNase-free water. RNA concentration and integrity was examined by Tape Station (Agilent). RNA was stored at −80 °C until use.

### RNA-Seq and differential gene expression analysis

RNA, extracted from EF3030 cultures grown in either THY or CSE on two independent occasions, was sequenced. Libraries were prepared using the TruSeq Stranded Total RNA with Ribo-Zero Bacteria kit (Illumina) to deplete bacterial rRNA. Sequencing was then performed on the Illumina HiSeq. 4000 platform in 2 × 75 bp reads (total read counts for THY samples 68,717,994 and 99,576,470; CSE samples 67,387,854 and 91,871,848). Because the EF3030 genome has not been fully sequenced, reads were mapped to the most closely related complete pneumococcal genome assembly available in the NCBI database at the time of analysis (CGSP14, serotype 14, accession no. NC_010582, which shared four of seven multi-locus sequence type alleles with EF3030) using Bowtie 2^57^. Differential expression of the capsular polysaccharide locus was assessed by mapping RNA-Seq reads to the serotype 19F capsule locus. A counts table was constructed using htseq-count^58^ to determine the number of reads that mapped to each gene from each condition (CSE versus THY). Differentially expressed genes were then identified from the counts table using the Voom/LIMMA package^59,60^, with data normalized for read depth and library size. Genes were defined as differentially expressed in CSE if the log_2_(fold change) in the CSE condition was greater than 1 or less than −1 and a false discovery rate cutoff of <0.05.

### Quantitative reverse transcription polymerase chain reaction (qRT-PCR)

To validate RNA-Seq data, a selection of differentially expressed genes identified by RNA-Seq were also tested using qRT-PCR. Pneumococcal cultures were once again incubated in THY or CSE and the RNA extracted. This RNA was used for first strand cDNA synthesis using the iScript™ cDNA Synthesis Kit (Bio-Rad). For each sample 2.5 ng RNA was added and incubated in a thermocycler under the following protocol: 5 min at 25 °C, 30 min at 42 °C and then 5 min at 85 °C. The cDNA was then stored at −20 °C until required. cDNA was used for qPCR in duplicate reactions using the GoTaq® qPCR Master Mix (Promega) as per the manufacturer’s instructions. Each reaction contained 2 µl of cDNA (or nuclease free water for the no template control) and 0.2 µM of forward and reverse primers. Primer sequences are provided in Table S1. Reactions were run on the Mx3005P qPCR system (Agilent Technologies) under the following conditions: 2 min at 95 °C followed by 40 cycles of 15 sec at 95 °C and 1 min at 60 °C. Non-specific amplification was assessed by performing a dissociation curve at the end of the run (1 min at 95 °C, 30 sec at 60 °C and then 30 sec at 95 °C). Analysis of each gene for changes in its expression in CSE relative to THY cultures was performed using the 2^−ΔΔCt^ relative quantification method^61^. The *gyrA* gene was used as a reference gene as described previously^62^.

### Pneumococcal adherence assay

The ability of CSE-exposed pneumococci to adhere to A549 lung epithelial cells was evaluated using an adherence assay. A549 cells were seeded overnight in a 24 well tray at 1.5 × 10^5^ cells per well. EF3030 cultures (incubated in either THY or CSE) were harvested by centrifugation and resuspended to ~1.5 × 10^8^ CFU/ml in 0.85% saline. Seeded A549 cells were washed with PBS prior to adding ~1.5 × 10^6^ CFU/ml pneumococci (or saline as a negative control) to duplicate wells. The tray was subject to centrifugation at 114 × *g* for 3 min before incubating at 37 °C, 5% CO_2_ for 1 h. Non-adherent bacteria were removed by washing the wells in PBS three times. Epithelial cells were subsequently lysed by the addition of 0.1% digitonin (Sigma-Aldrich) to each well and incubated at 37 °C, 5% CO_2_ for 5 min. Following incubation, cell-associated pneumococci were resuspended in THY and viable counting was conducted. The percentage of remaining pneumococci was calculated by comparing the number of remaining pneumococci to the starting inoculum added to the wells.

### Sensitivity to cell lysis

Bacterial cultures incubated in either THY or CSE were prepared as above. Cultures were then washed twice with PBS and resuspended in fresh THY media. To these cultures, 0.005% Triton X-100 was added and were incubated at 37 °C, 5% CO_2_. At 0, 15 and 30 min after the addition of Triton X-100, aliquots were taken for viable counting, and results used to calculate percent survival at each time point.

### Hydrophobicity assay

To assess cell surface hydrophobicity, the microbial adhesion to hydrocarbons assay was performed as described previously^63^. Pneumococcal cultures that had been incubated in either THY and CSE media were washed twice in PBS. At this point, an aliquot was taken for viable counts. Hexadecane was added to the bacterial suspensions and were mixed in a 3:1 ratio by vortexing for 2 min, followed by incubation at room temperature for 30 min. After incubation, an aliquot was taken from the aqueous phase for viable counts. This was used to calculate the proportion of pneumococci remaining in the aqueous phase.

## Electronic supplementary material

Supplementary Information

## Data Availability

Raw RNA-Seq data are available from the corresponding author upon reasonable request.

## References

[1] Wong J, Magun BE, Wood LJ (2016). Lung inflammation caused by inhaled toxicants: a review. Int. J. Chron. Obstruct. Pulmon. Dis..

[2] Arcavi L (2004). Cigarette smoking and infection. Arch. Intern. Med..

[3] Jones LL (2011). Parental and household smoking and the increased risk of bronchitis, bronchiolitis and other lower respiratory infections in infancy: systematic review and meta-analysis. Respir. Res..

[4] Fujioka K, Shibamoto T (2006). Determination of toxic carbonyl compounds in cigarette smoke. Environ. Toxicol..

[5] Bernhard D, Rossmann A, Wick G (2005). Metals in cigarette smoke. IUBMB Life.

[6] Kulkarni R (2012). Cigarette smoke increases *Staphylococcus aureus* biofilm formation via oxidative stress. Infect. Immun..

[7] Huang R (2014). Effects of nicotine on *Streptococcus gordonii* growth, biofilm formation, and cell aggregation. Appl. Environ. Microbiol..

[8] Antunes MB (2012). Molecular basis of tobacco-induced bacterial biofilms. Otolaryngol. Neck Surg..

[9] Semlali A, Killer K, Alanazi H, Chmielewski W, Rouabhia M (2014). Cigarette smoke condensate increases *C*. *albicans* adhesion, growth, biofilm formation, and *EAP1*, *HWP1* and *SAP2* gene expression. BMC Microbiol..

[10] Bagaitkar J (2011). Tobacco smoke augments *Porphyromonas gingivalis - Streptococcus gordonii* biofilm formation. PLoS One.

[11] Bagaitkar J (2010). Tobacco upregulates *P*. *gingivalis* fimbrial proteins which induce TLR2 hyposensitivity. PLoS One.

[12] McEachern EK (2015). Analysis of the effects of cigarette smoke on staphylococcal virulence phenotypes. Infect. Immun..

[13] Almirall Jordi, González Carlos A., Balanzó Xavier, Bolíbar Ignasi (1999). Proportion of Community-Acquired Pneumonia Cases Attributable to Tobacco Smoking. Chest.

[14] Nuorti JP (2000). Cigarette smoking and invasive pneumococcal disease. N. Engl. J. Med..

[15] Garcia-Vidal C (2010). Pneumococcal pneumonia presenting with septic shock: host- and pathogen-related factors and outcomes. Thorax.

[16] Shen P (2016). Cigarette smoke attenuates the nasal host response to *Streptococcus pneumoniae* and predisposes to invasive pneumococcal disease in mice. Infect. Immun..

[17] Voss M (2015). Cigarette smoke-promoted acquisition of bacterial pathogens in the upper respiratory tract leads to enhanced inflammation in mice. Respir. Res..

[18] Phipps JC (2010). Cigarette smoke exposure impairs pulmonary bacterial clearance and alveolar macrophage complement-mediated phagocytosis of *Streptococcus pneumoniae*. Infect. Immun..

[19] Hussey SJK (2017). Air pollution alters *Staphylococcus aureus* and *Streptococcus pneumoniae biofilms*, antibiotic tolerance and colonisation. Environ. Microbiol..

[20] Cockeran R (2014). Exposure of a 23F serotype strain of *Streptococcus pneumoniae* to cigarette smoke condensate is associated with selective upregulation of genes encoding the two-component regulatory system 11 (TCS11). Biomed Res. Int..

[21] Mutepe ND (2013). Effects of cigarette smoke condensate on pneumococcal biofilm formation and pneumolysin. Eur. Respir. J..

[22] Lee Changhan, Park Chankyu (2017). Bacterial Responses to Glyoxal and Methylglyoxal: Reactive Electrophilic Species. International Journal of Molecular Sciences.

[23] Begg SL (2015). Dysregulation of transition metal ion homeostasis is the molecular basis for cadmium toxicity in *Streptococcus pneumoniae*. Nat. Commun..

[24] Baliarda A (2003). Potential osmoprotectants for the lactic acid bacteria *Pediococcus pentosaceus* and *Tetragenococcus halophila*. Int. J. Food Microbiol..

[25] Kappes RM, Kempf B, Bremer E (1996). Three transport systems for the osmoprotectant glycine betaine operate in *Bacillus subtilis*: Characterization of OpuD. J. Bacteriol..

[26] Lucht JM, Bremer E (1994). Adaptation of *Escherichia coli* to high osmolarity environments: Osmoregulation of the high-affinity glycine betaine transport system ProU. FEMS Microbiol. Rev..

[27] Martín-Galiano AJ (2005). Transcriptional analysis of the acid tolerance response in *Streptococcus pneumoniae*. Microbiology.

[28] Martin-Galiano AJ, Ferrandiz MJ, De la Campa AG (2001). The promoter of the operon encoding the F0F1 ATPase of *Streptococcus pneumoniae* is inducible by pH. Mol. Microbiol..

[29] Moscoso M, García E, López R (2006). Biofilm formation by *Streptococcus pneumoniae*: Role of choline, extracellular DNA, and capsular polysaccharide in microbial accretion. J. Bacteriol..

[30] Kloosterman TG, Witwicki RM, Van Der Kooi-Pol MM, Bijlsma JJE, Kuipers OP (2008). Opposite effects of Mn2+ and Zn2+ on PsaR-mediated expression of the virulence genes *pcpA*, *prtA*, and *psaBCA* of *Streptococcus pneumoniae*. J. Bacteriol..

[31] Ogunniyi AD (2010). Central role of manganese in regulation of stress responses, physiology, and metabolism in *Streptococcus pneumoniae*. J. Bacteriol..

[32] Ding F (2009). Genome evolution driven by host adaptations results in a more virulent and antimicrobial-resistant *Streptococcus pneumoniae* serotype 14. BMC Genomics.

[33] Khan MN, Sharma SK, Filkins LM, Pichichero ME (2012). PcpA of *Streptococcus pneumoniae* mediates adherence to nasopharyngeal and lung epithelial cells and elicits functional antibodies in humans. Microbes Infect..

[34] Grigg J (2012). Cigarette smoke and platelet-activating factor receptor dependent adhesion of *Streptococcus pneumoniae* to lower airway cells. Thorax.

[35] Steinmoen H, Knutsen E, Håvarstein LS (2002). Induction of natural competence in Streptococcus pneumoniae triggers lysis and DNA release from a subfraction of the cell population. Proc. Natl. Acad. Sci. USA.

[36] Guiral S, Mitchell TJ, Martin B, Claverys J-P (2005). From The Cover: Competence-programmed predation of noncompetent cells in the human pathogen *Streptococcus pneumoniae*: Genetic requirements. Proc. Natl. Acad. Sci..

[37] Eldholm V, Johnsborg O, Haugen K, Ohnstad HS, Havarstein LS (2009). Fratricide in *Streptococcus pneumoniae*: contributions and role of the cell wall hydrolases CbpD, LytA and LytC. Microbiology.

[38] Kausmally L, Johnsborg O, Lunde M, Knutsen E, Håvarstein LS (2005). Choline-binding protein D (CbpD) in Streptococcus pneumoniae is essential for competence-induced cell lysis. J. Bacteriol..

[39] Gosink KK, Mann ER, Guglielmo C, Tuomanen EI, Masure HR (2000). Role of Novel Choline Binding Proteins in Virulence of Streptococcus pneumoniae. Infect. Immun..

[40] Wei H, Håvarstein LS (2012). Fratricide is essential for efficient gene transfer between pneumococci in biofilms. Appl. Environ. Microbiol..

[41] Oggioni MR (2006). Switch from planktonic to sessile life: A major event in pneumococcal pathogenesis. Mol. Microbiol..

[42] Marks, L. R., Reddinger, R. M. & Hakansson, A. P. High levels of genetic recombination during nasopharyngeal carriage and biofilm formation in *Streptococcus pneumoniae*. *MBio* **3** (2012). 10.1128/mBio.00200-12 PMC3448161 23015736

[43] Stevens KE, Chang D, Zwack EE, Sebert ME (2011). Competence in *Streptococcus pneumoniae* is regulated by the rate of ribosomal decoding errors. MBio.

[44] Slager J, Kjos M, Attaiech L, Veening JW (2014). Antibiotic-induced replication stress triggers bacterial competence by increasing gene dosage near the origin. Cell.

[45] Gagne AL (2013). Competence in Streptococcus pneumoniae is a response to an increasing mutational burden. PLoS One.

[46] Zhang Y-M, Rock CO (2009). Transcriptional regulation in bacterial membrane lipid synthesis. J. Lipid Res..

[47] Fozo EM, Quivey RG (2004). The *fabM* gene product of *Streptococcus mutans* is responsible for the synthesis of monounsaturated fatty acids and is necessary for survival at low pH. J. Bacteriol..

[48] Kovács M (2006). A functional *dlt* operon, encoding proteins required for incorporation of D-alanine in teichoic acids in Gram-positive bacteria, confers resistance to cationic antimicrobial peptides in *Streptococcus pneumoniae*. J. Bacteriol..

[49] Majchrzykiewicz JA, Kuipers OP, Bijlsma JJE (2010). Generic and Specific Adaptive Responses of *Streptococcus pneumoniae* to Challenge with Three Distinct Antimicrobial Peptides, Bacitracin, LL-37, and Nisin. Antimicrob. Agents Chemother..

[50] Nelson AL (2007). Capsule enhances pneumococcal colonization by limiting mucus-mediated clearance. Infect. Immun..

[51] Shenoy AT, Orihuela CJ (2016). Anatomical site-specific contributions of pneumococcal virulence determinants. Pneumonia.

[52] Throup JP (2002). A genomic analysis of two-component signal transduction in *Streptococcus pneumoniae*. Mol. Microbiol..

[53] Gómez-Mejia Alejandro, Gámez Gustavo, Hammerschmidt Sven (2018). Streptococcus pneumoniae two-component regulatory systems: The interplay of the pneumococcus with its environment. International Journal of Medical Microbiology.

[54] Haas W, Kaushal D, Sublett J, Obert C, Tuomanen EI (2005). Vancomycin stress response in a sensitive and a tolerant strain of *Streptococcus pneumoniae*. J. Bacteriol..

[55] Laan M, Bozinovski S, Anderson GP (2004). Cigarette smoke inhibits lipopolysaccharide-induced production of inflammatory cytokines by suppressing the activation of activator protein-1 in bronchial epithelial cells. J. Immunol..

[56] Andersson B (1983). Identification of an active disaccharide unit of a glycoconjugate receptor for pneumococci attaching to human pharyngeal epithelial cells. J. Exp. Med..

[57] Langmead B, Salzberg SL (2012). Fast gapped-read alignment with Bowtie 2. Nat. Methods.

[58] Anders S, Pyl PT, Huber W (2015). HTSeq-A Python framework to work with high-throughput sequencing data. Bioinformatics.

[59] Law CW, Chen Y, Shi W, Smyth G (2014). K. voom: precision weights unlock linear model analysis tools for RNA-seq read counts. Genome Biol..

[60] Ritchie ME (2015). limma powers differential expression analyses for RNA-sequencing and microarray studies. Nucleic Acids Res..

[61] Livak KJ, Schmittgen TD (2001). Analysis of relative gene expression data using real-time quantitative PCR and the 2−ΔΔCT method. Methods.

[62] Pettigrew MM (2014). Dynamic changes in the *Streptococcus pneumoniae* transcriptome during transition from biofilm formation to invasive disease upon influenza A virus infection. Infect. Immun..

[63] Rosenberg M, Gutnick D, Rosenberg E (1980). Adherence of bacteria to hydrocarbons: A simple method for measuring cell-surface hydrophobicity. FEMS Microbiol. Lett..

