# Supplementary material for: The transcriptomic response of Streptococcus pneumoniae following exposure to cigarette smoke extract
Source: Sci Rep. 2018 Oct 24;8:15716. doi: 10.1038/s41598-018-34103-5 (PMC6200755; doi:10.1038/s41598-018-34103-5)
Supplement: Supplementary file 1 — Supplementary Information [file 41598_2018_34103_MOESM1_ESM.docx]

**Supplementary Information**

The transcriptomic response of *Streptococcus* *pneumoniae* following exposure to cigarette smoke extract

Sam Manna, Alicia Waring, Angelica Papanicolaou, Nathan E. Hall, Steven Bozinovski, Eileen M. Dunne and Catherine Satzke

Supplementary Figure S1: Effect of CSE concentration on pneumococcal viability. Log-phase EF3030 cultures were exposed to various concentrations of CSE (50-400%, with 100% defined as one cigarette drawn through 10 ml THY medium) or THY (0%) for 45 min and then viable counts performed to enumerate the number of viable pneumococci. Data presented are from three independent experiments and displayed as the median with the interquartile range, and analyzed by Mann-Whitney test.

Supplementary Figure S2: qRT-PCR analysis examining differential expression of the *ply* operon in response to CSE over time. Log-phase pneumococci were exposed to CSE or THY control media for 5, 10, 15, 30 or 45 min prior to RNA extraction. Data were normalized to the *gyrA* gene using the 2-ΔΔCt method. Data are presented as the mean fold change ± standard deviation in cultures incubated in CSE relative to those incubated in THY control media

Table S1. List of primers used in this study for qRT-PCR analysis.

| Gene | Sequence (5’ to 3’) | Reference |
| --- | --- | --- |
| *gyrA* | Forward: CAATATGCTCGCTATCCAA  Reverse: GACGAACAACCACTTCTT | ^1^ |
| SPCG_RS00380 | Forward: GGAGATTACCATCATCATTTAGC  Reverse: GTGCGTCAACTTCTTGTGC | This study |
| SPCG_RS09490 | Forward: GATTGGACAATGTGGTCAGC  Reverse: GGGTTTGGTGAGTTTCTAGG | This study |
| SPCG_RS09520 | Forward: GCAAGATTTTAGCGGTTTTATGG  Reverse: CACTTCTTTCTGGGACTTGC | This study |
| SPCG_RS00385 | Forward: AAGGAGATCGTGACTGTGC  Reverse: TCTAGGCGTTCCTTTTGAGC | This study |
| SPCG_RS10300 | Forward: CAATTGCTCACGCTTCAACC  Reverse: GACCGCACGTTCAAAATACC | This study |
| SPCG_RS10625 | Forward: ATTATCAGCGTGCTTTTCTTGC  Reverse: CCCATCTGCTTGTAACTTGC | This study |
| SPCG_RS09065 | Forward: AGCGCAGTTTCTAGGATGG  Reverse: ATCCAAACCTGCTTCCAAGC | This study |
| SPCG_RS02210 | Forward: GATGATGGTGCAGTTGTTGG  Reverse: GACGGCCAAATTTCTCAGC | This study |
| SPCG_RS09950 | Forward: CTACCCGATGAGTTTGTTGTT  Reverse: TCCAGGATAGAGGCGACT | ^2^ |
| SPCG_RS11330 | Forward: GTTCGGTTGGGATTATCATCG  Reverse: ATCAACTCATCTCGCTCAGG | This study |
| SPCG_RS02155 | Forward: ATGTCATCGGTAAGGCTCC  Reverse: CCTTGTGGAAGCGTTTATGC | This study |
| SPCG_RS09945 | Forward: AAGGCTATCGTTGCTCTCC  Reverse: TCTTTTGGACGTCTTCGTCG | This study |

Table S2. Descriptive statistics of data displayed in Fig 2, fold change expression of genes in the presence of cigarette smoke.

|  | Strain | | | | | | | |
| --- | --- | --- | --- | --- | --- | --- | --- | --- |
|  | EF3030 | | | | PMP1287 | | | |
| Gene | Mean | Standard deviation | Lower 95% CI of mean | Upper 95% CI of mean | Mean | Standard deviation | Lower 95% CI of mean | Upper 95% CI of mean |
| SPCG_RS00380 | 50.28 | 14.46 | 14.36 | 86.19 | 10.94 | 1.76 | 6.58 | 15.31 |
| SPCG_RS09490 | 35.99 | 31.27 | -41.68 | 113.70 | 43.79 | 21.55 | -9.74 | 97.32 |
| SPCG_RS09520 | 4.93 | 1.29 | 1.73 | 8.13 | 4.08 | 0.33 | 3.27 | 4.89 |
| SPCG_RS00385 | 13.03 | 1.19 | 10.08 | 15.99 | 8.06 | 0.35 | 7.19 | 8.94 |
| SPCG_RS10300 | 1.49 | 0.27 | 0.83 | 2.16 | 6.30 | 1.93 | 1.51 | 11.09 |
| SPCG_RS10305 | 1.51 | 0.32 | 0.71 | 2.30 | 5.34 | 3.40 | -3.10 | 13.78 |
| SPCG_RS10625 | 1.85 | 0.62 | 0.31 | 3.39 | 0.54 | 1.69 | -3.67 | 4.74 |
| SPCG_RS09065 | 3.53 | 0.70 | 1.80 | 5.27 | 2.86 | 0.43 | 1.79 | 3.94 |
| SPCG_RS02210 | -14.80 | 8.00 | -34.66 | 5.07 | -8.58 | 8.63 | -30.02 | 12.85 |
| SPCG_RS09950 | -30.10 | 1.60 | -34.07 | -26.12 | -21.45 | 17.58 | -65.11 | 22.22 |
| SPCG_RS11330 | -2.64 | 0.65 | -4.24 | -1.03 | -2.01 | 0.30 | -2.75 | -1.26 |
| SPCG_RS02155 | -4.14 | 1.02 | -6.67 | -1.62 | -2.22 | 2.94 | -9.52 | 5.08 |
| SPCG_RS09945 | -3.56 | 2.32 | -9.32 | 2.20 | -0.70 | 1.61 | -4.69 | 3.29 |

References

1. Grohs, P. *et al.* Molecular basis for different levels of *tet*(M) expression in *Streptococcus pneumoniae* clinical isolates. *Antimicrob. Agents Chemother.* **56,** 5040–5045 (2012).

2. Gupta, R., Shah, P. & Swiatlo, E. Differential gene expression in *Streptococcus pneumoniae* in response to various iron sources. *Microb. Pathog.* **47,** 101–109 (2009).
